# Léiomyome vasculaire de l'avant bras: présentation d'un cas clinique et revue de la literature

**DOI:** 10.11604/pamj.2014.19.222.5381

**Published:** 2014-10-28

**Authors:** Erraji Moncef, Kharraji Abdessamad, Abassi Najib, Abdeljawad Najib, Hicham Yacoubi

**Affiliations:** 1 Unité de Chirurgie Orthopédique et Traumatologique, Centre Hospitalier Universitaire d'Oujda, Maroc

**Keywords:** Léiomyome vasculaire, tumeur, membre supérieur, traitement chirurgical, Vascular leiomyoma, tumor, upper limb, surgical treatment

## Abstract

Le léiomyome est une tumeur bénigne vasculaire qui peut se présenter sous la forme d'une masse localisée; cependant le diagnostic ne peut en être clinique. Le diagnostic définitif repose sur la réalisation de multiples examens d'imagerie radiologique et sur le résultat de l'examen histologique.une fois traitée par une excision large, passant en zone saine sur le vaisseau, la tumeur à un faible taux de récidive. Nous en rapportant un cas de patiente ayant un léiomyome siégeant au niveau de l'avant bras.

## Introduction

Les tumeurs vasculaires, en général sont rares. Le léiomyome correspond à une tumeur bénigne prenant origine dans le muscle lisse. [1]. Elles sont traitées par une excision large, sans recours à une thérapie adjuvante. Nous rapportons le cas de patiente ayant un léiomyome de l'avant bras.

## Patient et observation

**Figure 1:**
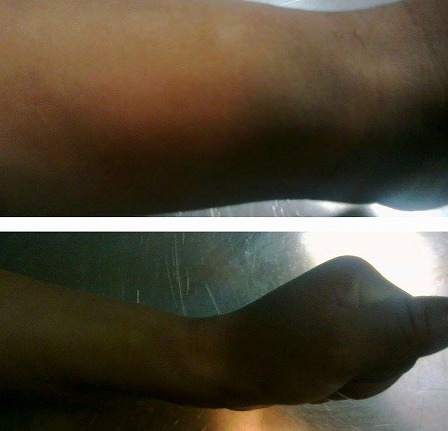
Aspect clinque d'une masse au niveau de l'avant bras

Mme K.H 30 ans, sans antécédents pathologiques, s'est présentée dans notre unité de chirurgie traumato-orthopédie pour une tuméfaction de la face postérieure du bord radial de l'avant bras gauche. A l'examen clinique, la masse était sous-cutanée de consistance ferme, d'environ 1 cm, bien limitée et douloureuse à la palpation. Elle était mobile par rapport aux deux plans. Les pouls périphériques étaient perçus et la patiente ne présentait ni varicosité ni oedème de l'avant bras (Figure 1).

**Figure 2:**
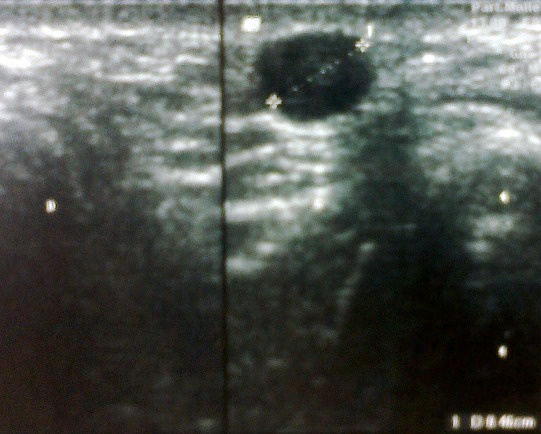
Image échographique montrant un nodule de 10mm sous cutané

**Figure 3:**
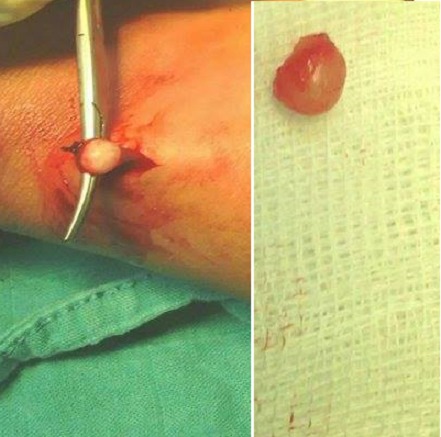
Exérèse chirurgical de la masse

**Figure 4:**
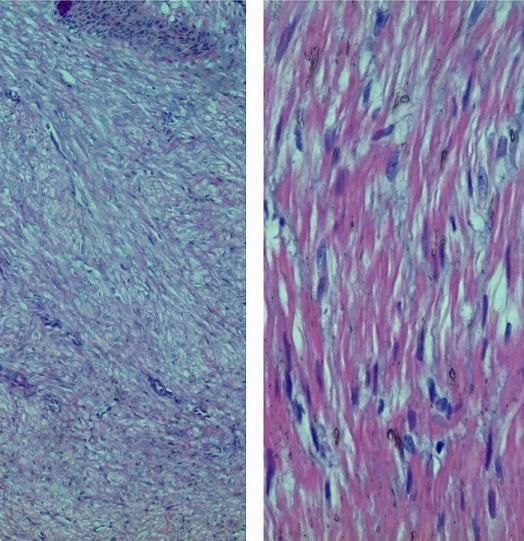
Aspect histologique, noter l'aspect de la tumeur à la périphérie de la lumière veineuse

**Figure 5:**
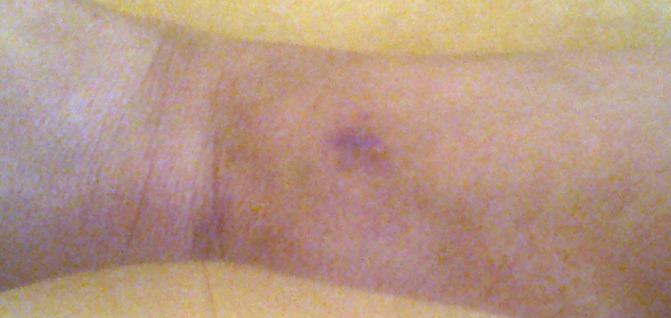
Pas de récidive après un recul de 12 mois

Les radiographies standards étaient normales. L’échographie des parties molles objectivait un nodule sous-cutané hétérogène de 10 mm, en continuité avec une veine superficielle (Figure 2). Les diagnostics de tumeurs glomiques, d'un shwanome d'un nerf superficiel ou d'une thrombose veineuse superficielle ont été suspectés. Une exérèse chirurgicale a été réalisée sous anesthésie locale. Il s'agissait d'une tumeur ronde rosée au dépend de la paroi d'une veine superficielle (Figure 3). L'examen anatomopathologique a montré une tumeur faite de cellules musculaires s'enroulant autour de la lumière veineuse en faveur d'un léiomyome de la paroi veineuse, sans aucun signe de malignité (Figure 4). A 12 mois de recul, il n'existait aucun signe de récidive local ou régional (Figure 5).

## Discussion

Dés 1937, Stout [2] distinguait le léiomyome vasculaire (ou angiomyome des anatomopathologistes) des léiomyomes cutanés. Les léiomyomes vasculaires se rencontrent plus fréquemment chez la femme avec une sex-ratio de 1,7/1 [3]. La tumeur peut survenir à tout âge mais le plus souvent après 40 ans. Elle est exceptionnelle chez le patient jeune ou âgé. L’âge moyen de l'apparition, le même pour les deux sexes, est de 47 ans [3, 4]. Les léiomyomes veineux peuvent se présenter comme une tuméfaction localisée et parfois sensible le long du vaisseau intéressé. Ils peuvent être révélés par une thrombose veineuse profonde, [5] bien que l’évolution tumorale lente permette habituellement le développement d'une circulation collatérale adéquate, ce qui rend ce mode de présentation plus inhabituel. [6] La taille de la lésion est généralement inférieure à 1 cm [7]. Elles siègent essentiellement au membre inférieur. Seuls Yasutaka et Akihiko en rapportaient un cas rare de léiomyome vasculaire géant de l'avant bras associé à des calcifications. L'atteinte osseuse est exceptionnelle et siégerait volontiers dans les localisations digitales [8].

Une série de Hachisuga et al rapporte que la localisation au niveau de l'avant bras survient que dans 2 % des cas, associés à des calcifications. [3] Microscopiquement, ils se composent de faisceaux entrelacés de cellules musculaires lisses entourant une lamina soulignée de cellules endothéliales normales. La douleur associée à ces tumeurs est supposée être mediée par les terminaisons nerveuses localisées au sein de la tumeur et dans la capsule, en réponse à des médiateurs mastocytaires ou à l’étirement mécanique. [9] Ces tumeurs sont réputées prendre plus souvent origine au niveau des veines que des artères et ont été rapportées au sein d'autres vaisseaux dont la veine basilique, la veine mésentérique supérieure et la veine iliaque externe. [9] L'origine réelle des léiomyomes vasculaires reste cependant controversée: naissent elles de la paroi vasculaire elle même ou du tissu conjonctif perivasculaire et ont elles un potentiel de transformation ? [10] On sait maintenant que leur diagnostic repose sur la mise en évidence au microscope d'un panaché de muscle lisse et d’éléments vasculaires. La majorité des publications concluent que le léiomyome vasculaire ne dégénère pas et ne récidive pas après exérèse chirurgicale. Cependant, deux auteurs relatent l'observation d'un cas d'une récidive locale [9]: ces deux récidives montraient en plus une dégénérescence maligne; il s'agissait probablement d'emblée d'un léiomyosarcome: quoiqu'il en soit, la confirmation histologique n'est obtenue que par l'exérèse chirurgicale qui reste indispensable.

## Conclusion

Le léiomyome vasculaire est une tumeur rare. Son diagnostic doit être évoqué devant le tableau clinique d'un nodule douloureux, isolé, ayant évolué lentement. La tumeur est deux fois plus fréquente chez la femme. Son pronostic est à la bénignité. Après exérèse chirurgicale le léiomyome vasculaire ne récidive pas.
